# High-throughput toxicity screening of novel azepanium and 3-methylpiperidinium ionic liquids†

**DOI:** 10.1039/d0ra03107k

**Published:** 2020-06-16

**Authors:** Angela L. Tether, Garry Laverty, Alberto V. Puga, Kenneth R. Seddon, Brendan F. Gilmore, Stephen A. Kelly

**Affiliations:** The QUILL Research Centre, School of Chemistry and Chemical Engineering, Queen's University Belfast BT9 5AG UK; Biofunctional Nanomaterials Group, School of Pharmacy, Queen's University Belfast BT9 7BL UK; Departament d’Enginyeria Química, Universitat Rovira i Virgili Avinguda dels Països Catalans, 26 43007 Tarragona Spain stephen.kelly@qub.ac.uk +44 28 9097 2305; The Biofilm Research Group, School of Pharmacy, Queen's University Belfast BT9 7BL UK

## Abstract

Ionic liquids (ILs) have been employed as potentially environmentally friendly replacements for harmful organic solvents, but have also been studied for their use in bioelectrochemical applications, such as in microbial electrochemistry for bioenergy production, or in industrial biocatalysis. For these processes, low microbial toxicity is important and there is a growing need for microbial toxicology studies for novel ILs. In this study, we report initial toxicity data for novel ILs, based on azepanium and 3-methylpiperidinium cations. Agar disc diffusion assays are used, along with minimum inhibitory concentration (MIC) and minimum bactericidal concentration (MBC) determinations, to obtain rapid and inexpensive initial toxicity data for these novel ILs against *Escherichia coli* and *Staphylococcus epidermidis*. Many of the novel ILs characterised possess low microbial toxicity relative to well-studied ILs, highlighting their potential for further study in applications where this is a desirable property.

## Abstract 2

Ionic liquids have been employed as potentially environmentally friendly replacements for organic solvents, but have also been studied for their use in bioelectrochemical applications, such as bioenergy production, or in industrial biocatalysis.

## Graphical abstract

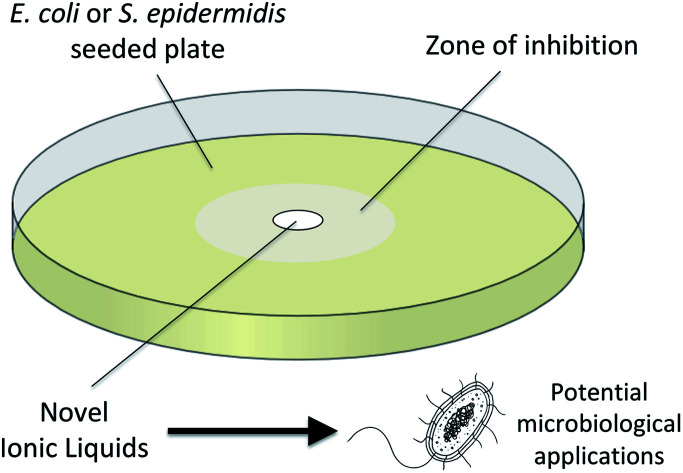

## 1. Introduction

The emergence of ionic liquids (ILs) as environmentally friendly replacements for harmful organic solvents and electrolytes continues to gain in popularity. Some early generation ILs, such as those containing some cations combined with [BF_4_]^−^, [PF_6_]^−^, and [NTf_2_]^−^, were initially touted as being ‘green’ due to properties like low volatility. Further studies however, showed them to be quite toxic to a number of organisms.^1^ This highlights the importance of early toxicological studies conducted alongside the synthesis of novel ILs. This is of paramount importance, whether they are to be used in industrial applications or in consumer products. The number of antimicrobial susceptibility studies involving ILs continues to increase, with ILs based on a number of functional groups, including imidazolium,^2–5^ alkylimidazolium lactates,^6^ oxygen-functionalised imidazolium esters,^7^ pyrrolidinium and piperidinium,^5^ quaternary ammonium ILs,^8–11^ morpholinium-based ILs,^12^ and phosphonium ILs.^13^

Novel ILs have evolved through various generations^14^ in order to improve their original use,^15,16^ as well as to reduce their toxicity. For example, first generation alkylimidazolium/alkylpyridinium chloroaluminate/halide ILs were developed for electrochemical applications,^17,18^ with [C_4_pyr][Al_2_Cl_7_]^−^ exhibiting both cyto- and ecotoxicity. Later generation ILs, including quaternary ammonium ILs, have been studied as electrolytes for batteries,^19^ with cholinium-based quaternary ammonium ILs possessing lower toxicity their predecessors.^8,20,21^ More recently, pyrrolidinium, piperidinium and azepanium-based ILs have been proposed as green electrolytes for battery systems, fuel cells and high voltage supercapacitors.^22–24^

ILs, which play important roles in electrochemical applications as highlighted above, can also be applied in microbial electrochemistry, such as in microbial fuel cells and supercapacitor manufacture.^25–28^ Ammonium-based polymer IL membranes have also been employed for wastewater treatment and bioenergy production using microbial fuel cells.^29,30^ Unlike for IL use as biocides, where high microbial toxicity is beneficial, low toxicity is desirable in microbial electrochemical and biocatalysis applications, so as not to inhibit the microorganisms involved in these biotransformations.

Similarly, low microbial toxicity is a desirable property for ILs involved in whole cell biocatalysis, in order to maintain the viability of the microorganism carrying out the biotransformation. ILs have been used to improve a number of biocatalytic processes, with their versatility allowing them to act as substitutes for both organic solvents and in aqueous two-phase systems, as well as possessing low flammability and negligible vapour pressure.^31,32^ ILs have been employed in a range of other applications involving microbes and their enzymes, such as biomass treatment and biodiesel production.^33,34^

In order to further much needed emphasis on early stage toxicity testing of novel ILs, this paper reports initial toxicity data for a new family of ILs based on azepanium and 3-methylpiperidinium cations. These are combined with the perfluorinated anions bis(trifluoromethane) sulfonimide ([NTf_2_]^−^), trifluoromethanesulfonate ([CF_3_SO_3_]^−^), and trifluoroacetate ([TFA]^−^), methylsulfate ([CH_3_SO_4_]^−^) and halide (I^−^ and Cl^−^) anions. Previously published data show moderate viscosities and remarkably wide electrochemical windows for these types of ILs, and suggests their potential for use as electrolytes, battery materials, or in synthetic media.^35,36^ In this study, agar disc diffusion assays are used, along with minimum inhibitory concentration (MIC) and minimum bactericidal concentration (MBC) determinations, to obtain rapid and inexpensive initial toxicity data for these novel azepanium and 3-methylpiperidinium ILs. Twenty-five different ILs were tested in all, including some halogenated ILs commonly used as starting materials for synthesis. Toxicity to bacterial strains *Escherichia coli* and *Staphylococcus epidermidis* were assessed, representing well studied examples of both Gram negative and Gram positive bacteria respectively.

## 2. Experimental

### 2.1. IL synthesis, disc preparation and sample quantification

The ILs used in this study were synthesized as described previously.^36,37^ The cations and anions used in this study are shown, along with their structures, in Table 1 and 2 respectively. IL purity was assessed by NMR spectroscopy, accurate mass ESI-MS, and CHNS elemental analysis.^36,37^ CHNS analysis is summarised in ESI (Table S1†). Grade 1 Whatman filter paper discs (6 mm diameter) were prepared using a metal hole punch and sterilised using a dry air oven. Sterile 1.5 mL microcentrifuge tubes were weighed both empty and with a sterile filter paper disc inside. 5 μL IL was added and a new weight recorded, before transferring the disc to the surface of an agar plate. The tube, along with residual IL was reweighed, and the amount of IL on the disc was determined from these measurements.

**Table 1:** Cations used in this study

Cation	Abbreviation	Structure
1-Methyl-1-(2-methoxyethyl)-pyrrolidinium	[MeOC_2_mpyrr]^+^	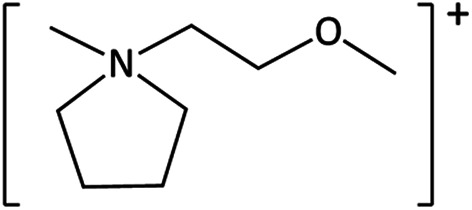
1-Methyl-1-butyl-3-methylpiperidinium	[C_4_mm_β_pip]^+^	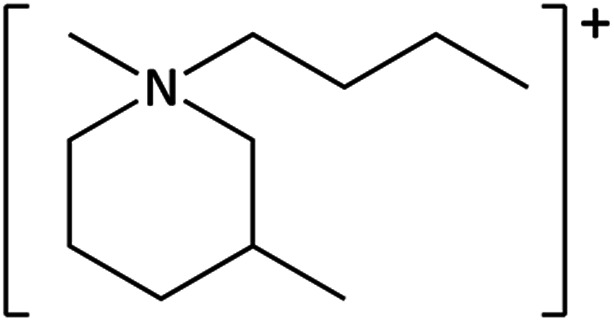
1-Methyl-1-hexyl-3-methylpiperidinium	[C_6_mm_β_pip]^+^	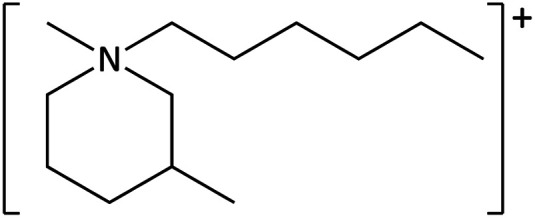
1-methyl-1-(2-methoxyethyl)-piperidinium	[MeOC_2_mpip]^+^	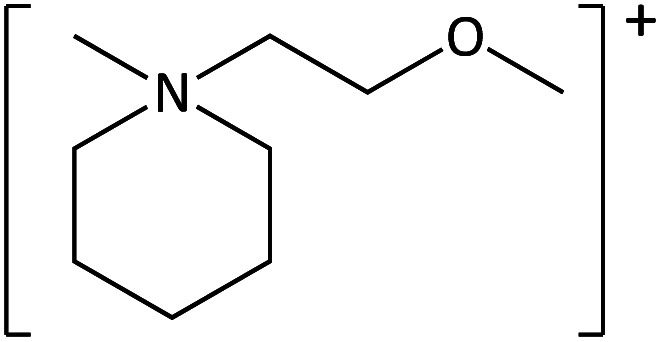
1-Methyl-1(2-methoxyethyl)-3-methylpiperidinium	[MeOC_2_mm_β_pip]^+^	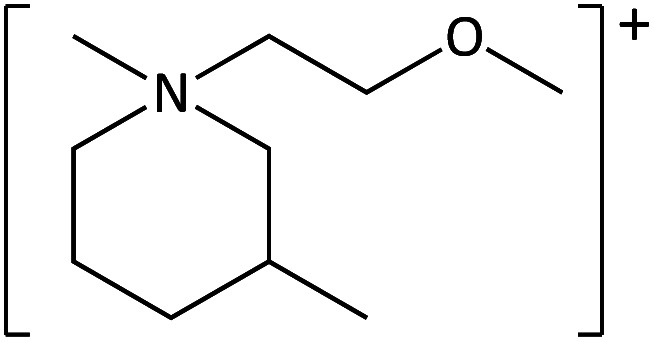
1-Methyl-1-[2-(2-methoxyethoxy)ethyl]-3-methylpiperidinium	[MeOC_2_OC_2_mm_β_pip]^+^	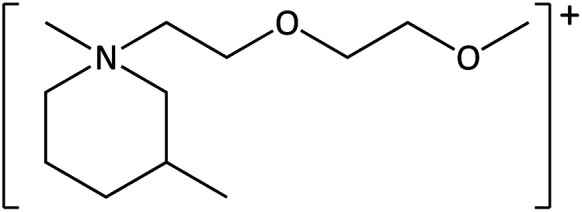
1-Methyl-1-butylazepanium	[C_4_mazp]^+^	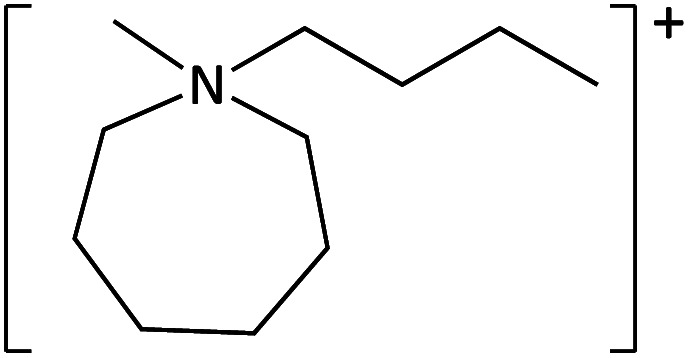
1-Methyl-1-hexylazepanium	[C_6_mazp]^+^	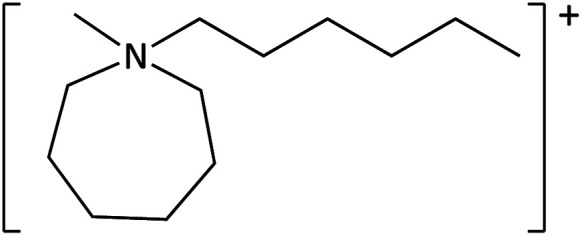
1-Methyl-1-(2-methoxyethyl)-azepanium	[MeOC_2_mazp]^+^	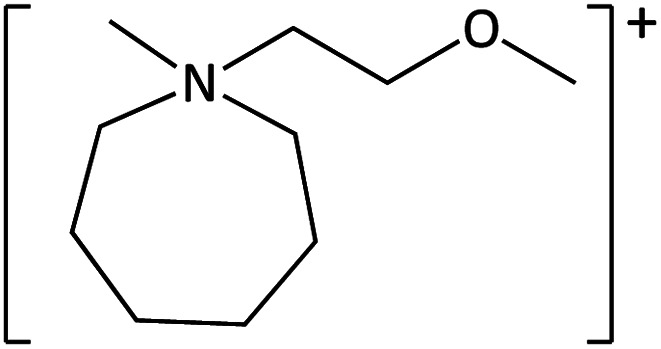
1-Methyl-1-[2-(2-methoxyethoxy)ethyl]-azepanium	[MeOC_2_OC_2_mazp]^+^	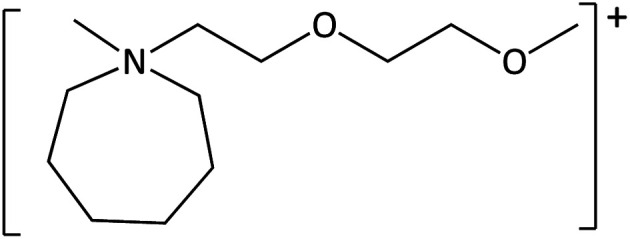

**Table 2:** Anions used in this study (Cl^−^ and I^−^ were also used but are not shown)

Name	Abbreviation	Structure
Methylsulfate	[MeSO_4_]^−^	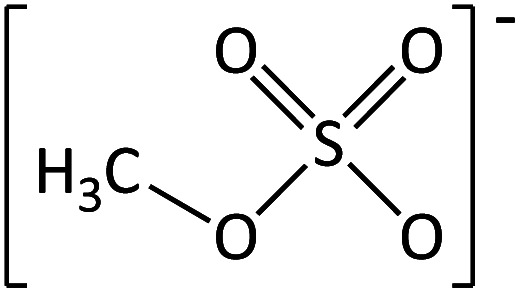
Bis(trifluoromethane) sulfonimide	[NTf_2_]^−^	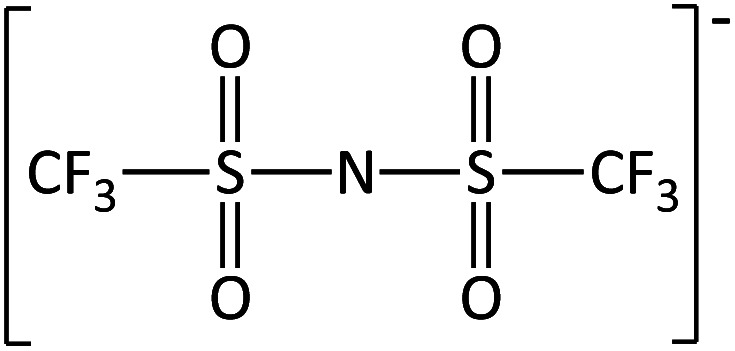
Trifluoromethane-sulfonate	[CF_3_SO_3_]^−^	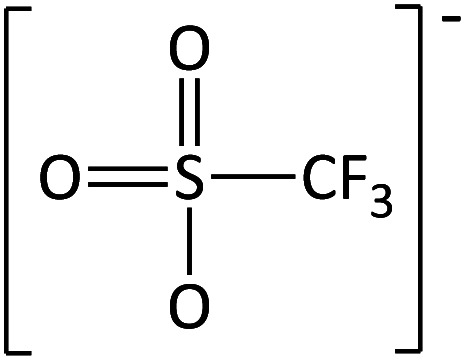
Trifluoroacetate	[TFA]^−^	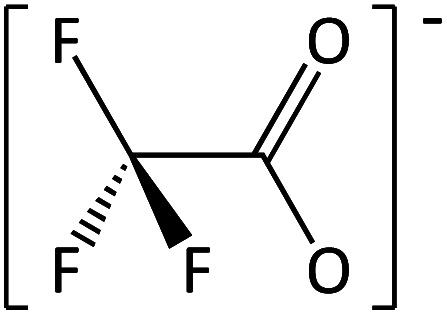

### 2.2. Agar disc diffusion tests

Agar disc diffusion tests were adapted from previously described reports.^20,38,39^*E. coli* 8196 and *S. epidermidis* ATCC 35984 were maintained on Mueller-Hinton Agar (MHA) and grown in Mueller-Hinton Broth (MHB). Bacterial suspension (50 μL) was spread on the surface of an MHA plate, with growth medium used as a negative control. Once dry, three IL discs were applied to the surface of each plate. Plates were allowed to incubate overnight at 37 °C.

### 2.3. MIC/MBC screening

*E. coli* 8196 cultures were grown overnight at 37 °C in MHB, followed by centrifugation at 3000 rpm for 15 min. The bacterial pellet was resuspended in MHB and adjusted to 1.5 × 10^8^ CFU mL^−1^ in Quarter-Strength Ringer's Solution. This suspension was diluted further in MHB to approximately 2 × 10^6^ CFU mL^−1^, as verified by a Miles and Misra total viable count.^40^ To determine MIC, doubling serial dilutions of ILs in MHB were set up in 96-well microtitre plates, starting with 100 μL IL and 100 μL inoculum. Positive controls consisted of 100 μL inoculum and 100 μL MHB, while negative controls consisted of 200 μL MHB only. Each set of tests and controls was set up with either four or eight replicates, depending on sample availability. The microtitre plate was incubated for 24 h at 37 °C and 95% relative humidity in a gyrorotary incubator. Following MIC determination, the MBC was determined by transferring 20 μL from each well that displayed no growth signs, onto MHA plates. MHA plates were then incubated in a stationary incubator at 37 °C overnight.

## 3. Results and discussion

### 3.1. Effect of anion selection

Characterization of these novel ILs shows a low toxicity profile for the majority of ILs tested, against both the Gram negative *E. coli* and Gram positive *S. epidermidis*. Many of the ILs tested produced very low or no inhibition of growth for either *E. coli* or *S. epidermidis*, as was the case with [MeOC_2_mpip][Cl], [MeOC_2_mpip][CF_3_SO_3_] [MeOC_2_mpip][TFA], [MeOC_2_mm_β_pip][I], [MeOC_2_OC_2_mm_β_pip][I], [MeOC_2_mazp][CF_3_SO_3_], [MeOC_2_mazp][TFA], [MeOC_2_OC_2_mazp][I], and [MeOC_2_OC_2_mazp][CF_3_SO_3_]. This low microbial toxicity, observed across both piperidinium and azepanium-based ILs, highlights the potential of these novel ILs for use in microbial applications.

Perhaps the greatest property in determining toxicity in the ILs tested was the selection of anion. The [TFA]^−^ and [CF_3_SO_3_]^−^ anions have been previously studied, with [CF_3_SO_3_]^−^ found to be quite toxic towards some cyanobacteria and diatoms when used alongside [C_4_mim]^+^.^41^ In one agar diffusion study, the [CF_3_SO_3_]^−^ anion was found to be as inhibitory towards *E. coli* as the equivalent IL bearing a [NTf_2_]^−^ anion.^42^ Also, when tested for cytotoxicity against IPC-81 murine (rat) cell lines, the [CF_3_SO_3_]^−^ anion, combined with [C_4_mim]^+^ exhibited higher toxicity than both [BF_4_]^−^ or [PF_6_]^−^.^43^ For the tests performed here, both the [TFA]^−^ and [CF_3_SO_3_]^−^ anions produced low or no inhibition when combined with the cations [MeOC_2_mpip]^+^, [MeOC_2_mm_β_pip]^+^, [MeOC_2_OC_2_mm_β_pip]^+^, [MeOC_2_OC_2_mazp]^+^, and [MeOC_2_mazp]^+^, with both *E. coli* and *S. epidermidis* (Table 3). Higher toxicity was observed for [TFA]^−^ for both bacteria when combined with [C_6_mazp]^+^, with zones of inhibition of 6.0 ± 0.5 and 7.3 ± 1.1 mm observed for *E. coli* and *S. epidermidis* respectively. The lack of side chain oxygenation in the [C_6_mazp]^+^ may account for this observation, as zones of inhibition values were higher for this cation, regardless of anion employed (Table 3).

**Table 3:** Toxicity of ionic liquids towards *E. coli* and *S. epidermidis* using agar disc diffusion^a^

Ionic liquid	*E. coli*		*S. epidermidis*	
	IL mass (mg)	Zone of inhibition (mm)	IL mass (mg)	Zone of inhibition (mm)
[C_4_mm_β_pip][I]	5.43 ± 0.35	2.0 ± 0.1	5.80 ± 0.35	0
[C_4_mm_β_pip][NTf_2_]	6.67 ± 2.41	5.6 ± 0.4	6.60 ± 0.89	2.8 ± 0.6
[C_6_mm_β_pip][I]	5.13 ± 0.23	5.9 ± 0.2	5.33 ± 0.96	6.6 ± 1.0
[C_6_mm_β_pip][NTf_2_]	5.00 ± 0.72	3.1 ± 0.4	6.23 ± 1.24	3.4 ± 0.4
[MeOC_2_mpip][Cl]	5.40 ± 0.10	0	5.37 ± 0.40	0
[MeOC_2_mpip][CF_3_SO_3_]	5.73^os^	0	—	—
[MeOC_2_mpip][TFA]	5.13^os^	0	4.57 ± 0.38	0
[MeOC_2_mm_β_pip][I]	6.00 ± 0.56	0.5^st^	6.23 ± 0.78	0
[MeOC_2_mm_β_pip][NTf_2_]	5.80^os^	8.0 ± 0.4	5.33 ± 2.58	3.8 ± 0.8
[MeOC_2_mm_β_pip][CF_3_SO_3_]	4.97 ± 0.58	0.9 ± 0.2	4.83 ± 1.14	0
[MeOC_2_mm_β_pip][TFA]	3.87 ± 0.42	0.6 ± 0.2	—	—
[MeOC_2_OC_2_mm_β_pip][I]	5.50 ± 0.26	0	6.07 ± 0.12	0
[MeOC_2_OC_2_mm_β_pip][NTf_2_]	5.10 ± 0.54	7.6 ± 0.3	6.10 ± 0.89	3.8 ± 0.5
[MeOC_2_OC_2_mm_β_pip][CF_3_SO_3_]	4.87 ± 0.42	0.8 ± 0.2	7.20 ± 1.30	0
[MeOC_2_OC_2_mm_β_pip][TFA]	7.83 ± 1.66	1.7 ± 0.4	4.77 ± 0.55	0
[C_4_mazp][NTf_2_]	4.17 ± 0.51	3.5 ± 0.4	5.03 ± 0.64	3.6 ± 0.3
[C_6_mazp][I]	6.27 ± 1.27	6.1 ± 0.4	6.30 ± 0.44	3.2 ± 1.3
[C_6_mazp][MeSO_4_]	4.73 ± 0.12	5.8 ± 0.5	5.03 ± 0.51	6.9 ± 1.3
[C_6_mazp][TFA]	4.47 ± 1.10	6.0 ± 0.5	4.80 ± 0.36	7.3 ± 1.1
[MeOC_2_mazp][NTf_2_]	5.57^os^	7.6 ± 0.3	7.07 ± 0.84	3.9 ± 0.6
[MeOC_2_mazp][CF_3_SO_3_]	4.70 ± 0.79	0.8 ± 0.2	5.40 ± 0.50	0
[MeOC_2_mazp][TFA]	4.13 ± 0.51	0.4 ± 0.2	6.00 ± 1.65	0
[MeOC_2_OC_2_mazp][I]	5.60 ± 0.46	0.5^st^	5.77 ± 0.64	0
[MeOC_2_OC_2_mazp][NTf_2_]	5.23^os^	7.7 ± 0.7	6.57 ± 0.80	4.0 ± 0.4
[MeOC_2_OC_2_mazp][CF_3_SO_3_]	4.80 ± 0.10	0.5^st^	11.4 ± 4.35	0

a ^os^ – one sample produced, ^st^ – single tests run.

**Fig. 1:**
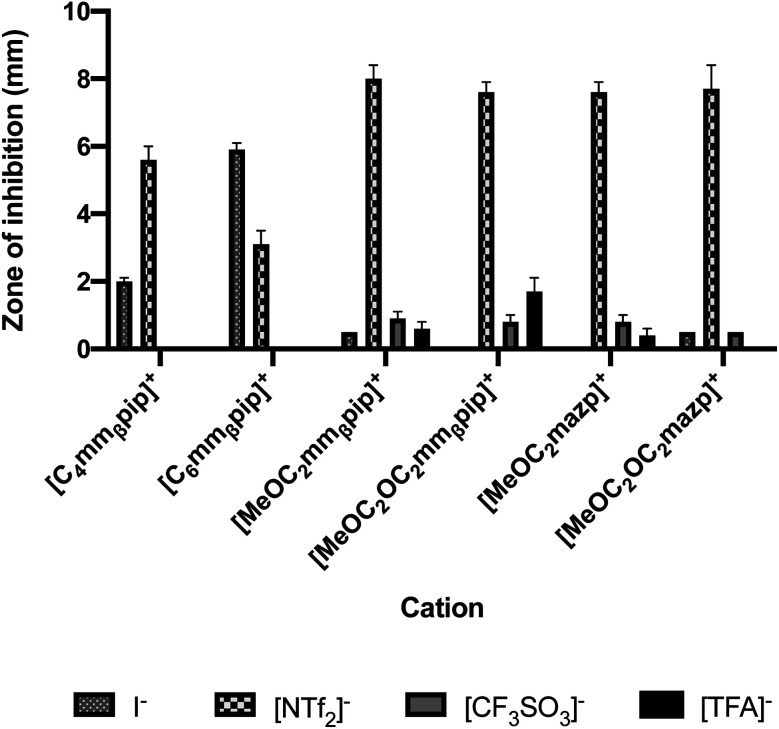
Effect of anion selection on toxicity towards *E. coli*, as measured by zone of inhibition using the agar disc diffusion test.

Incorporation of the bistriflimide anion [NTf_2_]^−^ resulted in a profound increase in toxicity, producing the greatest zones of inhibition across all cations it was combined with, with the exception of [C_6_mm_β_pip]^+^. Comparisons of toxicity against *E. coli* for different anion/cation combinations are shown in Fig. 1, with similar trends observed against *S. epidermidis*. These findings agree with previously published studies, which also reported high toxicity when [NTf_2_]^−^ was employed as an anion, particularly in combination with oxygen-functionalised cations.^44^

### 3.2. Effect of side chain oxygenation

The potential to tune IL toxicity through side chain oxygenation is interesting and could provide a route to non-toxic ILs with the desired physicochemical biological properties. Previous literature suggests oxygenation of side chains leads to a reduction in IL toxicity.^1,45–47^ This appears to be the case when [I]^−^ is chosen as an anion partner, with zones of inhibition for *E. coli* decreasing from 2.0 ± 0.1 to 0.5 mm respectively when [C_4_mm_β_pip][I] is oxygenated to [MeOC_2_mm_β_pip][I], from 6.1 ± 0.4 to 0.5 mm respectively when comparing [C_6_mazp][I] to [MeOC_2_OC_2_mazp][I], and from 5.9 ± 0.2 mm to 0 mm when comparing [C_6_mm_β_pip][I] to [MeOC_2_OC_2_mm_β_pip][I]. A similar trend is observed with *S. epidermidis* inhibition with the same IL pairs (Table 3).

This opposite effect is observed however when [NTf_2_]^−^ is selected as anion. In disc diffusion assays involving *E. coli*, the zone of inhibition, and therefore toxicity, increased from 5.6 ± 0.4 to 8.0 ± 0.4 mm when [C_4_mm_β_pip][NTf_2_] is compared with [MeOC_2_mm_β_pip][NTf_2_], and from 3.5 ± 0.4 to 7.6 ± 0.3 mm when [C_4_mazp][NTf_2_] is compared with [MeOC_2_mazp][NTf_2_]. Again, a similar trend is observed in the *S. epidermidis* experiments (Table 3). These results may be attributed to the combination of the hydrophobic bistriflimide anion with a more hydrophilic cation. As has been previously reported, it appears the more hydrophilic the cation, the more the anion will be “pulled” into aqueous solution, effectively increasing the concentration of the bistriflimide anion in the agar and making the IL more toxic.^39^

### 3.3. Other effects

Despite previous reports,^45,48^ an increase in the number of carbon atoms in the ring structure did not result in increased toxicity in this study, with comparable toxicities seen between piperidinium and azepanium-based ILs for both microorganisms. Additional methylation in the β-position of piperidinium-based ILs, whilst producing a small increase in the zone of inhibition for *E. coli*, does not appear to engender ILs with significant toxicity. Similar zones of inhibition were observed between [MeOC_2_mpip][TFA] (0 mm) and [MeOC_2_mm_β_pip][TFA] (0.6 ± 0.2 mm), and between [MeOC_2_mpip][CF_3_SO_3_] (0 mm) and [MeOC_2_mm_β_pip][CF_3_SO_3_] (0.9 ± 0.2 mm).

Physical properties of ILs, such as their solubilities in media, can also influence their toxicity. The water solubility of ILs is largely determined by the hydrophobicity of their constituent cations and anions,^49^ and this will also have had an effect on the ability of the ILs tested in this study to dissolve in water-based media and produce zones of growth inhibition.

Similar trends were observed between the two microorganisms used in this study, *E. coli* and *S. epidermidis*. ILs containing the [NTf_2_]^−^ anion appeared to be more toxic towards *E. coli* than *S. epidermidis*, with the exception of [C_4_mazp][NTf_2_] and [C_6_mm_β_pip][NTf_2_], where similar results were observed. Without these exceptions, zones of inhibition for [NTf_2_]^−^-containing ILs ranged from 5.6 ± 0.4 to 8.0 ± 0.4 mm for *E. coli*, and only 2.8 ± 0.6 to 4.0 ± 0.4 mm for *S. epidermidis*.

### 3.4. Determination of MIC and MBC values

**Fig. 2:**
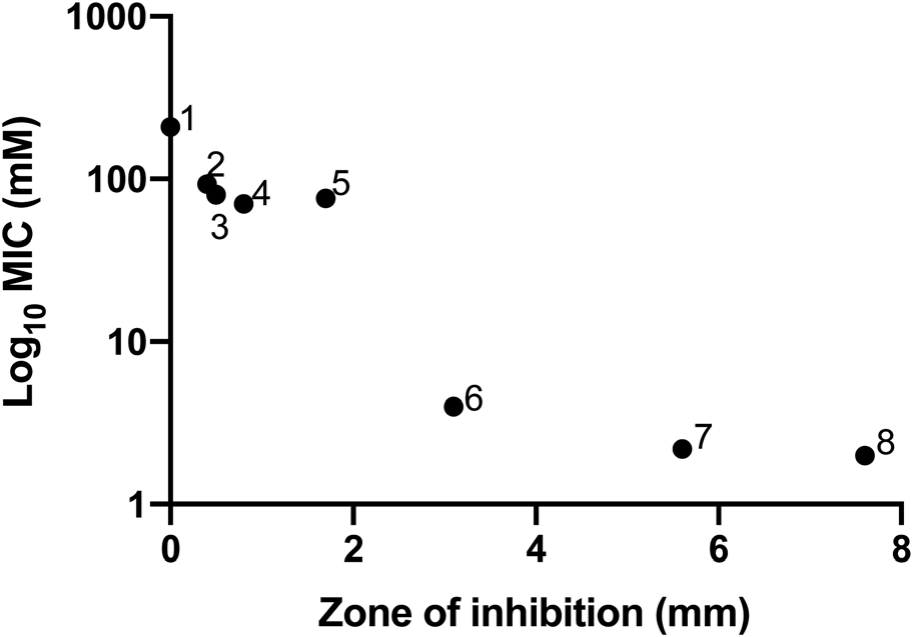
The relationship between toxicity values obtained for different ionic liquids towards *E. coli*, as measured using two different techniques. Toxicity values correlate closely between the agar disc diffusion assay (Zone of inhibition) and minimum inhibitory concentration determination assay (log_10_ MIC). The ionic liquids shown above are as follows: (1) [MeOC_2_mpip][TFA], (2) [MeOC_2_mazp][TFA], (3) [MeOC_2_OC_2_mazp][CF_3_SO_3_], (4) [MeOC_2_OC_2_mm_β_pip][CF_3_SO_3_], (5) [MeOC_2_OC_2_mm_β_pip][TFA], (6) [C_6_mm_β_pip][NTf_2_], (7) [C_4_mm_β_pip][NTf_2_], (8) [MeOC_2_OC_2_mm_β_pip][NTf_2_].

MIC and MBC values were determined for a range of ILs against *E. coli*, with the results shown in Table 4. In order to aid ease of comparison with published literature, in which MIC and MBC values are expressed using different units, results are shown in both μg mL^−1^ and mM. MIC values obtained ranged from <2.20 to 210 mM, with the lowest MIC values observed for ILs containing [NTf_2_]^−^ (<2.00, <2.00, and <2.20 for [C_6_mm_β_pip][NTf_2_], [MeOC_2_OC_2_mm_β_pip][NTf_2_] and [C_4_mm_β_pip][NTf_2_] respectively). These results are hardly surprising, given the high toxicity observed with bistriflimide anions in the disc diffusion assays. Indeed, there is a strong inverse correlation between zone of inhibition from the disc diffusion assay and MIC value, as shown in Fig. 2.

**Table 4:** MIC and MBC values for a range of ILs against *E. coli*

Ionic liquid	MIC		MBC	
	(μg mL^−1^)	(mM)	(μg mL^−1^)	(mM)
[C_4_mm_β_pip][NTf_2_]	<977	<2.20	<977	<2.20
[C_6_mm_β_pip][NTf_2_]	<977	<2.00	1950	4.00
[MeOC_2_mpip][TFA]	62 300	210.00	250000	845.00
[MeOC_2_OC_2_mm_β_pip][NTf_2_]	<977	<2.00	<977	<2.00
[MeOC_2_OC_2_mm_β_pip][CF_3_SO_3_]	24 720	70.00	24 720	70.00
[MeOC_2_OC_2_mm_β_pip][TFA]	25 040	76.02	50 000	152.04
[MeOC_2_mazp][TFA]	26 520	93.00	53 000	186.00
[MeOC_2_OC_2_mazp][CF_3_SO_3_]	27 780	80.00	55 560	160.00
[MeOC_2_OC_2_mazp][TFA]	25 000	76.00	50 000	150.00

In comparison to many previously reported MIC values, the novel ILs characterised in this study possess relatively low microbial toxicity, making them ideal for microbiological applications. For example, propargyl-functionalised piperidinium ILs, although designed for their toxicity, have been shown to have much lower MIC values against *E. coli* of 50 μg mL^−1^,^5^ whilst the ILs tested in this study have MICs many times that value. MIC values were also determined for a range of phosphonium bromide and chloride ILs against *E. coli* by Cieniecka-Rosłonkiewicz and co-workers, with values ranging from 0.01 to 0.02 mM for the bromides and 0.0025 to 0.113 mM for the chlorides.^13^ Work by the Gathergood group reported an MIC value of 16 μg mL^−1^ (0.0618 mM) for imidazolium-based IL 1-methyl-3-decylimidazolium against *E. coli*,^7^ and Pernak and co-workers reported MIC values of between 8 and 62.5 μg mL^−1^ for ammonium-based ILs against *E. coli*.^10^

These examples, across a range of IL classes, serve to highlight the relatively low toxicity of the ILs characterized in this study. Whilst low microbial toxicity is obviously undesirable for ILs if they are to employed as biocides, it is beneficial for their use in microbiological applications, such as biocatalysis, where it is critical for microbial function to be maintained.

## 4. Conclusions

In this study we have characterised the microbial toxicity of a number of novel ILs towards the well studied bacteria *E. coli* and *S. epidermidis*. Choice of anion had a particularly sizeable effect on toxicity, with [NTf_2_]^−^ in particular engendering high levels of toxicity across all ILs tested. Azepanium and piperidinium-based ILs had comparable toxicity profiles, and cations with oxygenated side chains exhibited reduced microbial toxicity with most anions. Low microbial toxicity towards both *E.* coli and *S.* epidermidis was observed for ILs with [MeOC_2_mpip]^+^ and [MeOC_2_OC_2_mm_β_pip]^+^ cations, highlighting in particular their potential for use in microbe-associated applications. Early characterisation of microbial toxicity is vital in determining the applicability of novel ILs for various uses. Comparisons of MIC values to previously reported ILs suggest those tested in this study have low overall toxicity, making them potential candidates for use in microbiological applications.

## Conflicts of interest

There are no conflicts to declare.

## Supplementary Material

RA-010-D0RA03107K-s001
