# Supplementary material for: High-throughput toxicity screening of novel azepanium and 3-methylpiperidinium ionic liquids
Source: RSC Adv. 2020 Jun 16;10(39):22864–70. doi: 10.1039/d0ra03107k (PMC9054695; doi:10.1039/d0ra03107k)
Supplement: RA-010-D0RA03107K-s001 [file RA-010-D0RA03107K-s001.pdf]

Electronic Supplementary Material (ESI) for RSC Advances.
This journal is © The Royal Society of Chemistry 2020

Supplementary Information

High-Throughput Toxicity Screening of Novel Azepanium and 3-Methylpiperidinium Ionic Liquids

Angela L. Tether, Garry Laverty, Alberto V. Puga, Kenneth R. Seddon, Brendan F. Gilmore, Stephen A. Kelly

Table S1. CHNS data for ionic liquids studied in this work.

Ionic liquid		C	H	N	S
[C$_4$mazp][NTf$_2$]	Found	34.7	5.1	6.2	14.3
	Calcd.	34.66	5.37	6.22	14.24
[C$_6$mazp][I]	Found	47.9	8.2	4.2	-
	Calcd.	48	8.68	4.31	-
[C$_6$mazp][TFA]	Found	57	8.9	4.45	
	Calcd.	57.86	9.06	4.5	
[MeOC$_2$mazp][TFA]	Found	50	7.6	4.95	-
	Calcd.	50.52	7.77	4.91	-
[MeOC$_2$mazp][CF$_3$SO$_3$]	Found	41.2	6.6	4.6	10.3
	Calcd.	41.11	6.9	4.36	10
[MeOC$_2$mazp][NTf$_2$]	Found	32.1	5.2	6.1	14.5
	Calcd.	31.86	4.9	6.19	14.17
[MeOC$_2$OC$_2$mazp][I]	Found	42.35	7.3	4.1	-
	Calcd.	41.99	7.63	4.08	-
[MeOC$_2$OC$_2$mazp][CF$_3$SO$_3$]	Found	42.4	6.8	4.2	8.75
	Calcd.	42.73	7.17	3.83	8.78
[MeOC$_2$OC$_2$mazp][NTf$_2$]	Found	34	5.2	5.8	13.2
	Calcd.	33.87	5.28	5.64	12.92
[C$_4$mm$_\beta$pip][I]	Found	44.2	7.6	4.7	-
	Calcd.	44.45	8.14	4.71	-
[C$_4$mm$_\beta$pip][NTF$_2$]	Found	34.9	5.4	6.2	14.9
	Calcd.	34.66	5.37	6.22	14.24
[C$_6$mm$_\beta$pip][I]	Found	48	8.4	4.3	-
	Calcd.	48	8.68	4.31	-
[C$_6$mm$_\beta$pip][NTF$_2$]	Found	37.6	5.9	5.9	13.3
	Calcd.	37.65	5.9	5.85	13.4
[MeOC$_2$mm$_\beta$pip][TFA]	Found	50	8.05	5.1	-
	Calcd.	50.52	7.77	4.91	-
[MeOC$_2$mm$_\beta$pip][CF$_3$SO$_3$]	Found	40.7	7.4	4.2	9.9
	Calcd.	41.11	6.9	4.36	9.98
[MeOC$_2$OC$_2$mm$_\beta$pip][I]	Found	41.7	7.95	4	-
	Calcd.	41.99	7.63	4.08	-

[$MeOC_2OC_2mm_\beta pip$][TFA]	Found	49.5	8.6	4.1	-
	Calcd.	51.05	7.96	4.25	-
[$MeOC_2OC_2mm_\beta pip$][CF_3SO_3]	Found	42.3	6.8	3.6	8.9
	Calcd.	42.73	7.17	3.83	8.78
[$MeOC_2OC_2mm_\beta pip$][NTf_2]	Found	34	5.4	5.2	13.3
	Calcd.	33.87	5.28	5.64	12.92
